# Poor sleep quality, COPD severity and survival according to CASIS and Pittsburgh questionnaires

**DOI:** 10.1038/s41598-023-45717-9

**Published:** 2023-10-31

**Authors:** Júlia Sampol, Marc Miravitlles, María Sáez, Mercedes Pallero, Gabriel Sampol, Jaume Ferrer

**Affiliations:** 1 Respiratory Department, Vall d’Hebron University Hospital, Barcelona, Spain; 2 Medicine Department, Universitat Autònoma de Barcelona (UAB), Barcelona, Spain; 3 Centro de Investigación Biomédica en Red de Enfermedades Respiratorias (CIBERES), Instituto de Salud Carlos III (ISCIIII), Barcelona, Spain; 4 Vall d’Hebron Institut de Recerca (VHIR), Barcelona, Spain

**Keywords:** Medical research, Epidemiology, Outcomes research

## Abstract

Poor sleep quality is frequent among COPD patients and it has been related to worse outcomes. The objective of this study was to compare the COPD and Asthma Sleep Impact Scale (CASIS) and the generic Pittsburgh Sleep Quality Index (PSQI) questionnaires as reliable tools for evaluating sleep quality and its relationship with COPD characteristics and survival. Stable COPD patients were prospectively evaluated. Anthropometric, sociodemographic, comorbidity, lung function and treatment data were collected. All patients completed CASIS and PSQI, mMRC dyspnea severity scale, COPD Assessment Test (CAT), sleep apnoea STOP-Bang and Hospital Anxiety and Depression Scale (HADS) questionnaires. Body mass index, airflow Obstruction, Dyspnea and Exacerbations (BODEx) index was calculated. Life status was determined after a mean follow-up of 3.7 (SD 1) years. We included 200 patients, 69.5% male, mean age 65.8 (9) years. Poor sleep was detected in 100 (50%) and 84 patients (42%) according to PSQI and CASIS questionnaires, respectively, with an agreement of 63%. Poor sleep was related to female gender, more severe dyspnea and worse BODEx, HADS and CAT scores according to both questionnaires. PSQI was associated to chronic pain or inferior urinary tract symptoms and CASIS to exacerbations, shorter walked distance in the 6-min walking test and treatment with oral corticosteroids or chronic oxygen. Thirty nine (19.5%) patients died during follow-up. Mortality was not associated to PSQI nor CASIS results. Unlike PSQI, CASIS is more related to COPD severity and its results are not influenced by comorbidities with known impact on sleep quality. In our sample, poor sleep quality was not associated with increased mortality.

## Introduction

Chronic Obstructive Pulmonary Disease (COPD) has a high prevalence all over the world^1^ and represents a major cause of mortality, morbidity, and health expenditure^2^. Among the multiple consequences of the disease, the deterioration of sleep quality is one of the most frequent^3^. This deterioration has been associated with greater severity of the underlying disease, poorer quality of life, more frequent exacerbations, an increased need for emergency care and higher mortality^4,5^. Despite its significance, however, sleep quality is not routinely assessed in patients with COPD. It has been evaluated as a potential therapeutic target, but the evidence obtained in this regard is limited^5^. For these reasons, it is essential to use well-designed questionnaires that allow accurate detection and quantification of sleep disturbances in COPD and can clarify the relationship between sleep quality and the clinical severity and prognosis of the disease^6^.

The questionnaire used most frequently to evaluate sleep quality in both clinical practice and research is the Pittsburgh Sleep Quality Index (PSQI)^7,8^. Initially developed as an instrument to distinguish between good and poor sleep quality in the general population, it has subsequently been used in patients with a variety of pathologies. It is widely used in patients with COPD, but assessments of its relationship with quality of life measures in these patients have shown contradictory results^9,10^. In addition, the relationship between PSQI and comorbidities and variables that reflect relevant aspects of the symptomatic burden of the disease has not been investigated^11,12^.

More recently, the COPD and Asthma Sleep Impact Scale questionnaire (CASIS)^13^ has been used in several studies^14,15^. Unlike the PSQI, the items of the CASIS questionnaire are focused on the detection of changes in sleep quality associated with the presence of respiratory problems. Easy to use in clinical practice, it appears to be a sensitive and specific tool for detecting the presence of changes in sleep quality secondary to COPD. However, these possible advantages have not yet been evaluated in relation to other questionnaires. Based on these considerations, in this study we evaluated the hypothesis that the PSQI and the CASIS questionnaires assess different aspects of sleep quality in stable COPD patients. To this end, we evaluated the results of both questionnaires regarding their concordance in the detection of poor/good sleep and performed a comparative analysis of their results with regard to the patients’ clinical characteristics and long-term mortality.

## Materials and methods

The study was carried out at the Pneumology Service and the Multidisciplinary Sleep Unit and approved by the Institutional Board Ethics Committee of our Hospital. It was conducted in accordance with the Declaration of Helsinki and written informed consent was obtained from all subjects involved in the study.

### Study design

The population in this prospective observational study comprised patients with stable COPD followed up at the outpatient clinic of our centre between January 2017 and December 2019. COPD was defined as post-bronchodilator forced expiratory volume in 1 s (FEV_1_) to forced vital capacity (FVC) ratio < 70% on spirometry performed in the six months prior to study entry, and stability as the absence of exacerbations or changes in treatment for at least four weeks prior to the baseline study assessment. All patients were smokers or ex-smokers of at least 10 pack-years. The study objectives were: (1) To analyse the concordance between the PSQI and CASIS questionnaires in determining poor/good sleep in stable COPD patients; (2) To compare the clinical characteristics and mortality during the study follow-up associated with sleep quality assessed by both questionnaires.

Patients were consecutively recruited, and those who did not meet the COPD stability criteria, had a previous diagnosis of obstructive apnoea or another sleep disorder (narcolepsy, periodic leg movements, shift work) or had active cancer or any other comorbidity in non-stable phase were excluded, as were those who were unable to understand the questionnaires administered.

### Baseline visit

A systematic review was performed of the hospital electronic medical records, including comorbidities and medications prescribed. The following variables were collected for each patient: (1) anthropometric and sociodemographic data (age, sex, body mass index); (2) smoking history (active or ex-smoker) and exposure factor (pack-years); (3) information on comorbidity according to the Charlson Index^16^ and the presence of osteoarticular pain or lower urinary tract symptoms (assessed by a specialist); (4) data on the diagnosis of COPD: degree of obstruction (FEV_1_ in mL and %), 6-min walking test (6MWT) (meters), exacerbations requiring corticosteroids and/or antibiotics in the last year. An exacerbator was defined as any patient with two or more moderate exacerbations (requiring treatment with antibiotics or oral corticosteroids) or one severe exacerbation (requiring hospitalization) in the last year; (5) chronic COPD treatment with short-acting β_2_-agonists, long-acting β_2_-agonists, short-acting muscarinic antagonists, long-acting muscarinic antagonists, inhaled corticosteroids, theophylline, roflumilast, azithromycin, chronic home oxygen therapy; and (6) use of anxiolytic or antidepressant treatment.

The following questionnaires were completed: (1) the modified Medical Research Council (mMRC) scale for the degree of dyspnea^17^; (2) the COPD assessment test (CAT) for health status^18^; (3) the STOP-Bang to screen for obstructive sleep apnoea^19^; (4) the Hospital Anxiety and Depression scale (HADS) for the presence of symptoms of anxiety-depression^20^; and (5) the Pittsburgh Sleep Quality Index (PSQI) and the COPD and Asthma Sleep Impact Scale (CASIS) on sleep quality. The CAT, HADS, PSQI and CASIS scales were self-administered. COPD severity was evaluated using the BODEx index^21^.

### Evaluation of sleep quality

#### Pittsburgh Sleep Quality Index (PSQI)

The PSQI is a self-administered questionnaire which assesses sleep quality over the last month. It includes 19 individual items which generate seven "component" scores: subjective sleep quality, sleep latency, sleep duration, habitual sleep efficiency, sleep disturbances, use of sleeping medication, and daytime dysfunction. A global score is calculated as the sum of these seven scores; patients with scores > 5 are classified as poor sleepers.

#### COPD and Asthma Sleep Impact Scale (CASIS)

The CASIS is a patient-reported 7-item questionnaire developed to assess sleep impairment associated with COPD and asthma symptoms over the last week. Five items assess the impact of breathing problems on falling asleep or staying awake during the day, and two items assess the impact of breathing problems on subjective sleep quality. The response options are scored on a Likert scale: (1) never; (2) rarely; (3) sometimes; (4) often; and (5) very often, and the last two items are reverse-scored. The sum of the scores on the seven items generates a total raw score which is then linearly transformed to a 0–100 total scale score. Higher scores indicate greater sleep impairment. There is no clearly defined cut-off point for the CASIS; we considered sleep to be of poor quality if the response of one or more of the items was > 2, on the grounds that having sleep impairment very often, often or sometimes during a period of a week can reasonably reflect sleep deterioration.

We also explored the relationship between the scores obtained on the CASIS and the PSQI and that of item 7 on the CAT questionnaire, which evaluates the presence or absence of sound sleep due to respiratory problems on a scale of 0 to 5. Specifically, we studied the difference in the scores of CAT item 7 between patients classified as good or poor sleepers by the CASIS and the PSQI.

### Follow up

The patients continued to be seen by their referral pulmonologist and their vital status was monitored until June 2022.

### Statistical analysis

Descriptive data are presented as n (percentage) for qualitative variables and mean standard deviation (SD) for quantitative ones. Comparisons between groups according to sleep quality were made using the t-test for unpaired data with quantitative variables and using Pearson’s Chi-square test with categorical variables. The agreement between both questionnaires was assessed using the concordance of their definitions of poor/good sleep and the Spearman’s correlation coefficient of their values.

The survival curves based on the results of the sleep questionnaires were estimated using the Kaplan–Meier method and compared using the log-rank test. Statistical significance was accepted for p < 0.05. Data analysis was carried out with Stata software (ver. 14.0; Stata Corporation, College Station, TX, USA).

### Ethics approval and consent to participate

The study was approved by the Ethics Committee of the Vall d’Hebron University Hospital [PR(AG)34/2017]. Written informed consent was obtained from all subjects involved in the study.

## Results

Table 1 shows the characteristics of the 200 patients included. They were mostly male (69.5%), with a mean age of 65.8 years (SD 9) and a body mass index (BMI) of 26.1 (SD 5) kg/m^2^. The patients had a smoking exposure factor of 48.7 (30.1) packs-year, and 27.5% were active smokers. The mean FEV_1_ was 50.3% (17.9) and 47% of patients were considered exacerbators.

**Table 1:** Demographic and clinical characteristics of COPD patients.

Descriptive data	Total
Age	65.8 (9)
Sex (female)	61 (30.5%)
BMI (kg/m^2^)	26.1 (5)
Smoking (current)	55 (27.5%)
Pack-years	48.7 (30.1)
Charlson	3.9 (1.7)
Osteoarticular pain	29 (14.5%)
Lower urinary tract symptoms	44 (22%)
Exacerbators	94 (47%)
BODEx	2.6 (2)
FEV_1_ mL	1411 (626)
FEV_1_%	50.3 (17.9)
6MWT (meters)	369 (83)
mMRC	1.4 (0.9)
CAT	13.9 (8.4)
PSQI	6.2 (4.1)
CASIS	14.8 (21.4)

Data are presented as mean (SD) or n (%).

*BMI* body mass index, *BODEx b*ody mass index, airflow Obstruction, Dyspnea and Exacerbations index, *FEV*_*1*_ forced expiratory volume in 1 s, *FEV*_*1*_*%* FEV_1_ percentage of predicted, *6MWT* 6 min walking test, *mMRC* modified Medical Research Council scale of dyspnea, *CAT* COPD assessment test, *PSQI* Pittsburgh Sleep Quality Index, *CASIS* COPD and Asthma Sleep Impact Scale.

**Figure 1:**
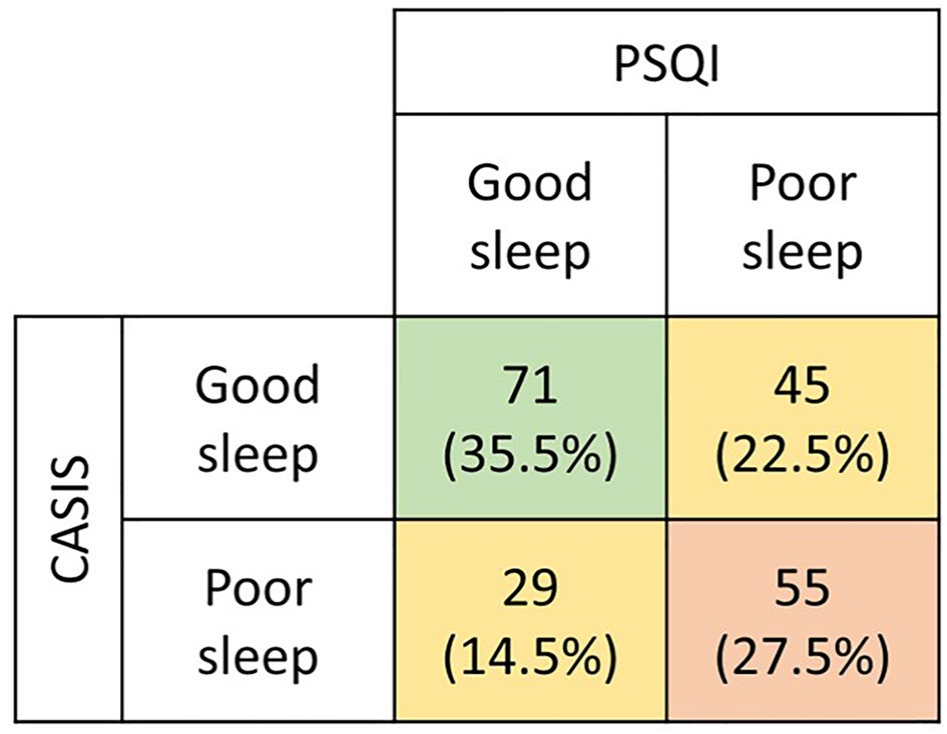
Good and poor sleepers according to PSQI and CASIS questionnaires.

The percentage of patients with poor sleep quality was 50% according to the PSQI and 42% according to the CASIS. The degree of agreement between the results of the two questionnaires was 63% (Fig. 1) and the Spearman’s correlation of their values was rho = 0.5 (p < 0.001).

Table 2 shows the comparative evaluation of the clinical characteristics and the results of the questionnaires according to the quality of sleep established by the PSQI and the CASIS.

**Table 2:** Demographic and clinical characteristics according to sleep quality evaluated by the PSQI and CASIS questionnaires.

Variables	PSQI Good Sleepers n = 100	PSQI Poor Sleepers n = 100	p	CASIS Good Sleepers n = 116	CASIS Poor Sleepers n = 84	p
Age	67.6 (8.2)	63.9 (9.4)	0.0038	66.7 (8.5)	64.5 (9.5)	0.0844
Sex (male)	76 (76%)	63 (63%)	0.046	89 (76.7%)	50 (59.5%)	0.009
BMI (kg/m^2^)	26.7 (5.2)	25.5 (4.7)	0.0719	26 (4.5)	26.2 (5.6)	0.7053
Smokers (current)	21 (21%)	34 (34%)	0.040	28 (24.1%)	27 (32.1%)	0.211
Pack-years	52.2 (32.7)	45.3 (27)	0.1068	48.3 (29.6)	49.3 (30.9)	0.8265
Charlson Index	4.1 (1.6)	3.7(1.7)	0.0877	3.9 (1.8)	3.9 (1.6)	0.8757
Osteoarticular pain	7 (7%)	22 (22%)	0.003	18 (15.5%)	11 (13.1%)	0.780
Lower urinary tract symptoms	16 (16%)	28 (28%)	0.041	28 (24.1%)	16 (19%)	0.391
Exacerbators	46 (46%)	48 (48%)	0.777	46 (39.7%)	48 (57.1%)	0.014
BODEx	2.3 (1.8)	2.9 (2.1)	0.0387	2.3 (1.9)	3.1 (1.9)	0.0047
FEV_1_ mL	1398 (595)	1425 (659)	0.7651	1465.8 (679.2)	1337.3 (540.1)	0.1525
FEV_1_%	50.3 (16.5)	50.3 (19.4)	0.9759	50.8 (18.3)	49.7 (17.5)	0.6744
6MWT (meters)	378 (76)	359 (91)	0.2296	387.1 (80.3)	347 (80.7)	0.0099
mMRC	1.2 (0.8)	1.6 (0.9)	0.0013	1.2 (0.8)	1.8 (0.8)	< 0.0001
CAT	10.4 (6.3)	17.3 (0.8)	< 0.001	10 (6.1)	19.2 (8.2)	< 0.0001
CAT item 7	0.3 (0.9)	1.5 (1.8)	< 0.001	0.2 (0.7)	1.9 (1.9)	< 0.0001
PSQI	3 (1.3)	9.3 (3.2)	< 0.001	4.9 (3.4)	7.9 (4.3)	< 0.0001
CASIS	6.9 (11.5)	22.8 (25.7)	< 0.001	1.6 (3.2)	33.1 (22.4)	< 0.0001
STOP-Bang	2.5 (1.6)	2.7 (1.7)	0.6104	2.7 (1.5)	2.5 (1.9)	0.3706
HADS_A	4.3 (3.5)	8.1 (4.5)	< 0.001	5 (3.9)	7.8 (4.7)	< 0.0001
HADS_D	3.5 (3.1)	6.8 (4.8)	< 0.001	3.6 (3.6)	7.2 (4.4)	< 0.0001

Data are presented as mean (SD) or n (%).

*BMI* body mass index, *BODEx* Body mass index, airflow Obstruction, Dyspnea and Exacerbations index, *FEV*_*1*_ forced expiratory volume in 1 s, *FEV*_*1*_*%* FEV_1_ percentage of predicted, *6MWT* 6 min walking test, *mMRC* modified Medical Research Council scale of dyspnea, *CAT* COPD assessment test, *PSQI* Pittsburgh Sleep Quality Index, *CASIS* COPD and Asthma Sleep Impact Scale, *HADS* Hospital Anxiety and Depression scale for the presence of symptoms of anxiety (HADS_A) and depression (HADS_D).

Poor sleep quality, assessed with both questionnaires, was related to female sex, a worse BODEx score, a higher degree of dyspnea, and worse score on the CAT and HADS questionnaires. Additionally, according to the PSQI, poor sleep quality was related to the presence of chronic osteoarticular pain or lower urinary tract symptoms. According to the CASIS, poor sleep quality was more frequent in patients defined as exacerbators and with a shorter distance walked in the 6MWT.

No differences were observed in the use of bronchodilator treatment or inhaled corticosteroids according to the sleep quality as measured by the questionnaires. However, in patients with poor sleep quality according to the CASIS, a greater use of oral corticosteroids (p: 0.009) and chronic home oxygen therapy (p: 0.005) was observed. In our sample, 24.6% of the patients regularly used anxiolytic and/or antidepressant drugs, with no differences between groups of good or poor sleepers.

**Figure 2:**
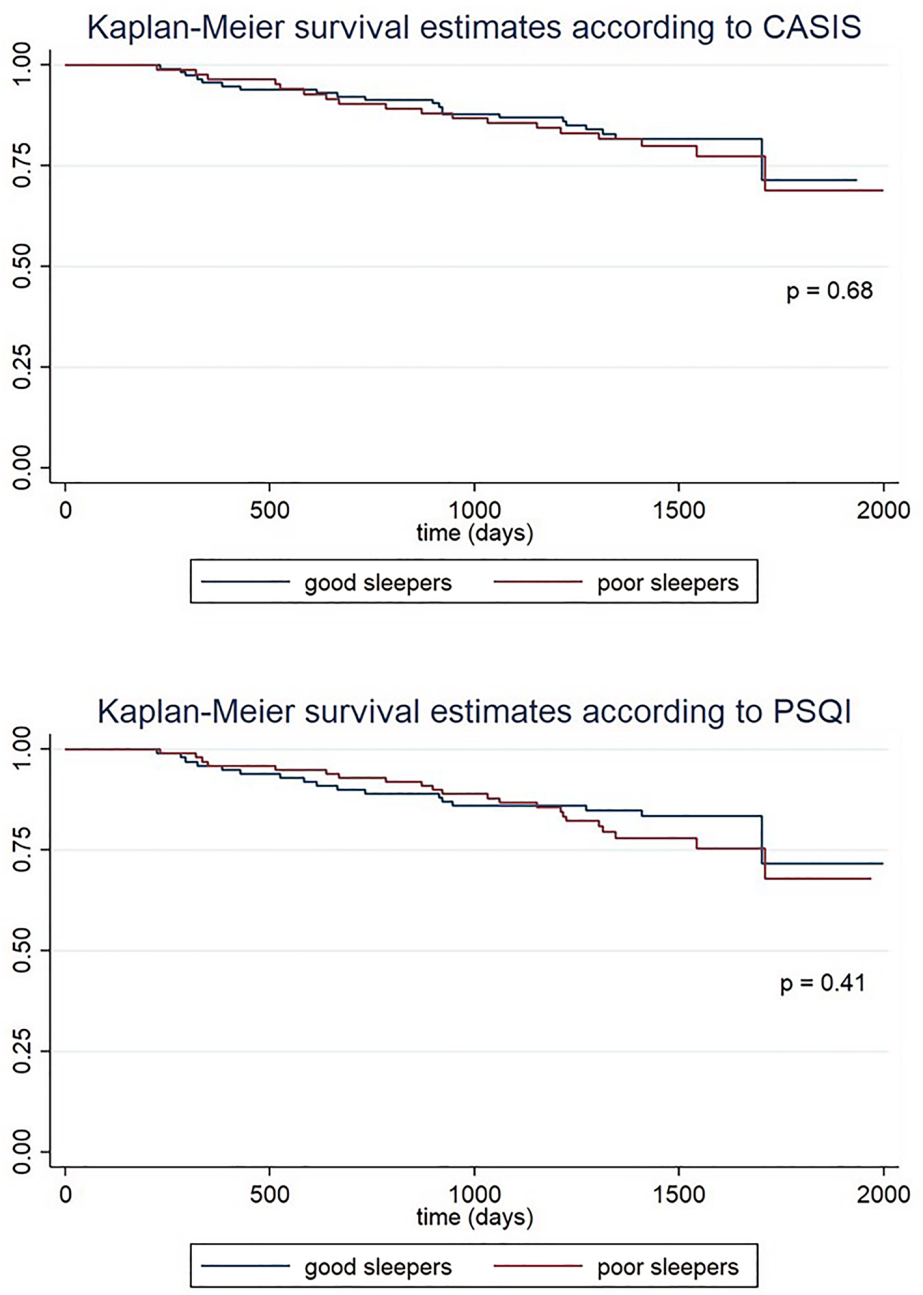
Kaplan–Meier survival curves according to sleep quality assessed by PQI or CASIS questionnaires.

Thirty-nine (19.5%) patients died during a mean follow-up of 3.7 (1) years. The main causes of death were respiratory (36%) and cancer (33%). In the comparative analysis (Fig. 2), we did not detect higher mortality of any cause in relation to poor sleep quality measured by the PSQI (log-rank test p: 0.4) or by the CASIS (log-rank test p: 0.7).

## Discussion

This study tested the hypothesis that the PSQI and the CASIS assess different aspects that may influence sleep quality, related or not to the characteristics of COPD. Our results seem to confirm this hypothesis since the overall concordance between the two questionnaires was only 63%; thus, more than a third of the patients showed discordant results depending on the questionnaire used. Clinical differences were detected in the variables associated with good/poor sleep in both questionnaires. The presence of poor sleep quality in the PSQI or CASIS was not associated with an increase in mortality at 3.7 years.

In agreement with previous reports, the definitions of poor sleep (according to both the PSQI classical criteria and the definition proposed in this work to evaluate the CASIS results) were associated with general variables such as female sex and higher scores on the anxiety and depression questionnaires^14,22,23^. Similarly, poor sleep quality assessed by both questionnaires was related to aspects of COPD severity, such as a higher degree of dyspnea and a worse CAT score. Most of these associations have been previously described elsewhere and confirm the idea that poor sleep quality is related to the severity of COPD^4,24^. Regarding the degree of obstruction and its relationship with sleep quality, the results in the literature are conflicting. In one study using the PSQI^25^, poor sleepers had a worse FEV_1_ but in two other studies no difference was detected^26,27^. As for the CASIS, its relationship with bronchial obstruction has been analysed in two studies^24,28^, which found an association between lower FEV_1_ values and higher questionnaire scores, but poor sleep quality could not be associated with a lower FEV_1_ because of the lack of a cut-off value. In our study, poor sleep quality defined by the two questionnaires was not associated with the degree of bronchial obstruction.

The relationship between sleep quality and certain COPD variables and other comorbidities differed depending on the questionnaire used. An important finding in this regard is that, unlike the PSQI, the CASIS was not related to non-respiratory problems that can disturb sleep, such as osteoarticular pain or lower urinary tract symptoms. These problems are common in the general adult population^29–31^ and may therefore introduce an element of confusion as to the cause of the sleep disturbances. Another noteworthy result is that only the CASIS was associated with a low value for the distance covered in the 6MWT, with the number of COPD exacerbations during the previous year and with chronic use of oral corticosteroids or chronic home oxygen therapy. The relationship between poor sleep quality and COPD exacerbations has been analysed in several previous studies. In general, when using the PSQI questionnaire, no association has been shown when adjusting for sleep-related morbidities^27,32^; using the CASIS, an association was detected in two previous studies^14,15^. Taken together, the data obtained suggest that poor sleep quality according to CASIS results is related to variables of COPD severity to a greater extent than the PSQI. Therefore, at a practical level, improving the treatment of COPD could be a strategy to improve sleep quality in patients classified as poor sleepers by the CASIS^33^.

The results of the CASIS questionnaire discussed in the previous paragraphs support the definition of poor/good sleep adopted in this study, which identified poor sleep in 42% of our patients. However, the clinical value of a questionnaire is increased if we have a cut-off value that allows dichotomizing patients. In the case of the CASIS questionnaire, we do not have this cut-off value. Ideally, the description of a cut-off value requires the analysis of the questionnaire results with respect to a gold-standard measure. However, it is known that we do not have objective markers of sleep quality, which continues being largely subjective and inconsistently quantifiable by current methods including polysomnography^34^. It should be noted that we found a mean value of the CASIS questionnaire in line with other studies^24,33^ but lower than that found by other authors^13,15,23,28^. The reasons for this discrepancy are not clear. We studied stable COPD patients and most of them reported none or very infrequent respiratory problems that interfered with sleep, and consequently the score obtained in the questionnaire was very low. Only 59% of our patients had a CAT ≥ 10, a value indicative of a medium or higher impact of COPD on health status and previously associated with a marked increase in the CASIS value^15^. Contrarily, a CAT ≥ 10 was present in 91%^15^ and 82%^28^ of other series that found mean CASIS values higher than ours. This allows us to speculate that the discrepancy in the CASIS values between the different studies could be related to the overall COPD severity perception from the patient's perspective. However, further studies on the clinical characteristics involved in the variability of CASIS are needed.

In the present study, neither the CASIS nor the PSQI value was related to mortality after a follow-up time of almost four years. The prognostic value of sleep quality had been studied in a previous study in a cohort of 98 patients with COPD^4^. Sleep quality (assessed using a different ad-hoc questionnaire) was associated with a higher number of exacerbations and higher all-cause mortality after a mean follow-up time of 2.4 years. The discrepancy on this point between that study and ours may lie in the type of questionnaire used, which makes comparison difficult. While Omachi et al.’s questionnaire focuses mainly on the presence of insomnia, the CASIS and the PSQI also cover other dimensions of sleep. Insomnia has previously been associated with mortality in the general population^35,36^ and this factor could explain the association detected by Omachi et al.

This study has some limitations. Firstly, its observational design means that no causal relationships can be inferred between sleep quality and the characteristics of patients with COPD. Regarding the comparison between the definitions of poor sleep quality provided by the two questionnaires, it should be borne in mind that the period of time covered by the two surveys differs; in the CASIS it is a week and in the PSQI it is a month. However, in the case of stable COPD patients, we do not believe that this difference could raise doubts about the results.

Strengths of the study that should be highlighted include its prospective design, the clinical profile of the patients with an exhaustive analysis of comorbidity and, especially, the follow-up time in terms of the mortality assessment.

## Conclusions

In conclusion, this study shows that the assessment of sleep quality using the CASIS questionnaire is not affected by non-respiratory comorbidities that do influence the PSQI. Likewise, the CASIS is related to variables reflecting COPD severity in a higher degree than the PSQI. Poor quality sleep assessed by the CASIS and the PSQI was not associated with an increase in 3,7-years mortality.

## Data Availability

The data of this study are available from the first author (julia.sampol@vallhebron.cat) upon reasonable request.

## Abbreviations

BMI: Body mass index
BODEx: Body mass index, airflow Obstruction, Dyspnea and Exacerbations index
CASIS: COPD and asthma sleep impact scale
CAT: COPD assessment test
COPD: Chronic obstructive pulmonary disease
FEV_1_: Forced expiratory volume in 1 s
FEV_1_%: FEV_1_ percentage of predicted
FVC: Forced vital capacity
HADS: Hospital anxiety and depression scale
mMRC: Modified Medical Research Council
PSQI: Pittsburgh sleep quality index
6MWT: 6-Minute walking test
SD: Standard deviation
